# Advanced Hybrid Nanocatalysts for Green Hydrogen: Carbon-Supported MoS_2_ and ReS_2_ as Noble Metal Alternatives

**DOI:** 10.3390/ijms26146640

**Published:** 2025-07-10

**Authors:** Maria Jarząbek-Karnas, Zuzanna Bojarska, Patryk Klemczak, Łukasz Werner, Łukasz Makowski

**Affiliations:** Faculty of Chemical and Process Engineering, Warsaw University of Technology, Waryńskiego 1, 00-645 Warsaw, Poland; maria.jarzabek.dokt@pw.edu.pl (M.J.-K.); patryk.klemczak.stud@pw.edu.pl (P.K.); lukasz.werner@pw.edu.pl (Ł.W.); lukasz.makowski.ichip@pw.edu.pl (Ł.M.)

**Keywords:** hydrogen evolution reaction, MoS_2_-based materials, rhenium disulfide nanoparticles, impinging jet reactor, wet chemical synthesis, synthesis of nanocatalysts, carbon nanomaterials, two-dimensional materials, PEM electrolysis

## Abstract

One of the key challenges in commercializing proton exchange membrane (PEM) electrolyzer technology is reducing the production costs while maintaining high efficiency and operational stability. Significant contributors to the overall cost of the device are the electrode catalysts with IrO_2_ and Pt/C. Due to the high cost and limited availability of noble metals, there is growing interest in developing alternative, low-cost catalytic materials. In recent years, two-dimensional transition metal dichalcogenides (2D TMDCs), such as molybdenum disulfide (MoS_2_) and rhenium disulfide (ReS_2_), have attracted considerable attention due to their promising electrochemical properties for hydrogen evolution reactions (HERs). These materials exhibit unique properties, such as a high surface area or catalytic activity localized at the edges of the layered structure, which can be further enhanced through defect engineering or phase modulation. To increase the catalytically active surface area, the investigated materials were deposited on a carbon-based support—Vulcan XC-72R—selected for its high electrical conductivity and large specific surface area. This study investigated the physicochemical and electrochemical properties of six catalyst samples with varying MoS_2_ and ReS_2_ to carbon support ratios. Among the composites analyzed, the best sample on MoS_2_ (containing the most carbon soot) and the best sample on ReS_2_ (containing the least carbon soot) were selected. These were then used as cathode catalysts in an experimental PEM electrolyzer setup. The results confirmed satisfactory catalytic activity of the tested materials, indicating their potential as alternatives to conventional noble metal-based catalysts and providing a foundation for further research in this area.

## 1. Introduction

Nowadays, one of the key challenges in the search for green alternatives to fossil fuels is the development of efficient and cost-effective methods of producing clean energy. In addition to solar, wind, and nuclear power, one of the most promising zero-carbon fuels is hydrogen. However, its commercial use requires the development of economically viable and efficient production technologies. Among them, water electrolysis powered by renewable sources enables the production of green hydrogen, a clean, sustainable fuel with no CO_2_ emissions, making it a key component of the future low-carbon energy landscape [1,2]. This study contributes to this goal by developing advanced hybrid nanocatalysts tailored for green hydrogen production.

Particular attention is currently being paid to hydrogen production via proton exchange membrane (PEM) electrolysis, an emission-free way of generating hydrogen. The water conversion produces only hydrogen and oxygen; however, the high cost of producing electrolyzers remains a major challenge. A key factor influencing their cost is the use of catalysts, which most often contain expensive precious metals such as platinum (Pt) and iridium (Ir), significantly increasing the overall production expenses [3]. Additionally, platinum is one of the rarest elements, and its extraction is concentrated in a few regions of the world, with geopolitical risks and potential disruptions to supply chains. As a result, this limits not only the availability of this raw material, but also the ability to scale PEM technology on a large scale. The work presented here proposes alternative catalysts based on more accessible elements such as molybdenum (Mo) and rhenium (Re), which can significantly reduce the costs and increase technological independence in hydrogen production.

There is an ongoing search for alternative elements that could replace expensive Pt and Ir-based catalysts in water electrolysis. However, in the case of the anodic oxygen evolution reaction, various catalysts have been tested, and those based on precious metals remained the most effective. Meanwhile, for the hydrogen evolution reaction, some of the most promising alternatives include tungsten (W) [4], molybdenum (Mo) [5], rhenium (Re) [6], and ruthenium (Ru) [7]. Catalysts based on two-dimensional transition metal dichalcogenides (2D TMDCs) are gaining particular attention [8]. The layered structure of TMDCs provides a high density of active catalytic centers, especially at defect edges and surfaces, where active reaction sites are exposed. These compounds exhibit unique catalytic properties comparable to those of noble metals, making them attractive candidates for HER applications [9]. Transition metal dichalcogenides can exist in different structural phases, resulting from the different coordination of the metal atoms. The most common two phases are 2H, characterized by triangular prismatic coordination of the metal atoms, and 1T, in which the metal atoms are surrounded in an octahedral fashion [10]. Both MoS_2_ and ReS_2_ in their natural state crystallize in the 2H phase, in which the layers are arranged in an ABA sequence. This structure promotes the formation of a large specific surface area, which translates into a large number of active sites. This is a key feature in catalytic applications including the hydrogen evolution reaction (HER). The 2H phase of TMDCs exhibits a number of advantages such a layered structure that allows for surface modification and the exposure of edges that are catalytically active, have high chemical and electrochemical stability, and a solid-state nature. These properties make 2H phase TMDCs a promising alternative as low-cost alternatives to platinum-based catalysts.

Molybdenum disulfide (MoS_2_) and rhenium disulfide (ReS_2_) are among the most widely studied TMDCs for electrolysis applications. MoS_2_ and ReS_2_ are considered promising catalysts for the hydrogen evolution reaction (HER) due to a combination of structural, electronic, and chemical properties that make them efficient, cost-effective alternatives to noble metals like platinum. To improve the electrical conductivity and dispersion of the catalyst nanoparticles, a Vulcan XC-72R carbon support may be used [10]. Vulcan XC-72R is a commonly used catalyst support due to its excellent chemical stability, large specific surface area, and high electrical conductivity. Thanks to its developed porosity, it enables the uniform deposition of catalyst nanoparticles such as MoS_2_ or ReS_2_, thereby increasing the number of active sites for electrochemical reactions [11].

Hybrid nanostructures composed of TMDCs and carbon supports represent an economically attractive solution for hydrogen production [12]. Due to their low cost, synergistic properties, and wide availability, these materials serve as highly effective catalysts in modern electrolyzers, thereby contributing to the advancement of more efficient and cost-effective hydrogen production technologies.

Herein, we study the influence of the carbon support (Vulcan XC72R content on the electrochemical properties of hybrid nanostructures based on widely available MoS_2_ and ReS_2_), a topic that has remained largely unexplored. These hybrid structures are synthesized via wet chemical synthesis using an impinging jet reactor [13,14,15]. Thanks to the use of a jet reactor for the catalyst production, we can influence their properties by controlling the flow of reactants, reaction temperature, and the amount of carbon support used. A detailed analysis of the morphology and structure of the produced materials was carried out using microscopic techniques to evaluate their structure and the distribution of nanoparticles on the support surface. In addition, the chemical composition was characterized, identifying the phases present and the degree of dispersion of the active components. The electrocatalytic properties of the tested catalysts under various experimental conditions were also evaluated to determine their potential toward hydrogen evolution reaction applications. Through a comprehensive structural and electrochemical characterization, we identified the most efficient hybrid structures for application in water PEM electrolysis.

The use of molybdenum and rhenium disulfide instead of platinum will not only significantly reduce the cost of production, but also opens up new perspectives for the development of cheaper and more accessible catalysts for efficient hydrogen production. This approach can contribute to the spread of hydrogen technologies and increase their competitiveness in the energy market. Despite the growing interest in 2D transition metal chalcogenides (2D TMDCs), the use of hybrid structures based on MoS_2_, ReS_2_, and carbon supports as catalytic materials in proton exchange membrane (PEM) electrolyzers remains largely unexplored. The present work represents one of the first approaches to experimentally evaluate these materials as potential substitutes for platinum in a real PEM system, which is an important part of the novelty of this study.

## 2. Results and Discussion

### 2.1. Physicochemical Characteristics

#### 2.1.1. Thermogravimetric Analysis

**Figure 1:**
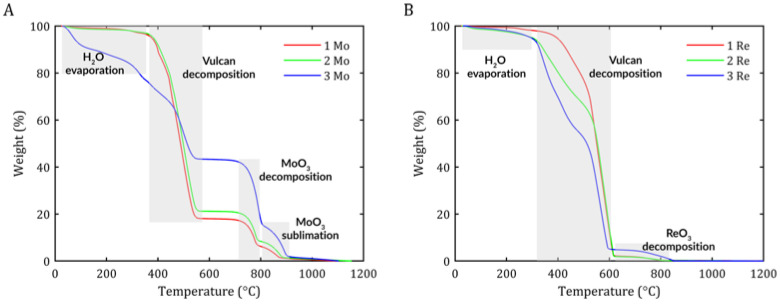
TGA analysis of samples 1 Mo, 2 Mo, and 3 Mo for MoS_2_/Vulcan (**A**) and 1 Re, 2 Re, and 3 Re for ReS_2_/Vulcan (**B**).

Thermogravimetric analysis (TGA) was performed to verify the purity of the sample and calculate the carbon content of the hybrid nanostructures. When analyzing the curves obtained for the MoS_2_/Vulcan samples (Figure 1A), several characteristic stages of mass loss could be observed. The first mass decrease occurred in the temperature range of 80–200 °C due to the removal of water absorbed in the sample. The next significant decrease in mass was observed in the 400–500 °C range, which can be attributed to the decomposition of carbon (Vulcan) and the oxidation of MoS_2_ to hexagonal-MoO_3_ (h-MoO_3_), and eventually phase transformation to α-MoO_3_. At 700 °C, MoO_3_ decomposed into lower oxides [16], whereas above 800 °C, the weight loss was related to the sublimation of α-MoO_3_ [17].

From the TGA results, the percentage carbon mass content was calculated as 79.06% (1 Mo), 87.4% (2 Mo), and 42.48% (3 Mo), and the approximate percentage molybdenum mass content was 16.6% (1 Mo), 20.37% (2 Mo), and 42.35% (3 Mo). In this case, it can be seen that a C/Mo ratio of 4.7 was achieved for sample 1 Mo, 3.6 for sample 2 Mo, and approximately 1 for sample 3 Mo. This means that with decreasing amounts of carbon added, the deposition of MoS_2_ on the surface of the support is more intense, resulting in a higher deposition of this compound in the sample.

Several decreases in mass could be observed when analyzing the TGA results for the ReS_2_-based samples (Figure 1B). The first decrease was observed in the temperature range of 80–200 °C, as for the MoS_2_ sample. This decrease was caused by the evaporation of water contained in the sample. The next mass decrease of about 40% was observed in the temperature range of 240–550 °C, most likely due to the decomposition of S_4_^2−^ polysulfide and the sublimation of Re_2_O_7_. The origin of Re_2_O_7_ is related to adsorbed ReO_4_, which can recombine at higher coverage levels and temperatures, leading to the formation of volatile Re_2_O_7_ [18,19]. Another mass loss of about 45% at 450–600 °C was probably caused by carbon decomposition from the sample. A final mass loss of 2–5% was likely caused by the sublimation of ReO_3_ [15].

From the TGA results, the percentage carbon mass content was calculated as 92.95% (1 Re), 92.10% (2 Re), and 88.06% (3 Re), and the approximate percentage rhenium sulfide mass content was 2.26% (1 Re), 2.03% (2 Re), and 5.18% (3 Re). As observed, the samples containing ReS_2_ showed a higher carbon content relative to the catalyst, which may indicate a less efficient synthesis reaction on the surface of the Vulcan support compared with the formation of MoS_2_.

#### 2.1.2. X-Ray Fluorescence Spectroscopy

X-ray fluorescence spectroscopy (XRF) measurements were also carried out to accurately determine the percentage compactness of specific elements in the samples. The results of the analysis of the MoS_2_/Vulcan samples are given in Table 1. Carbon was the main element in samples 1 Mo, 2 Mo, and 3 Mo. The carbon content in the samples was not proportional to the amount of carbon added at the beginning of the synthesis, which may be due to its effect on the course of MoS_2_ synthesis or to the increased penetration of MoS_2_ into the pores of the support used.

Table 1 shows the molar ratio S to Mo in all of the studied samples. In samples 3 Mo and 2 Mo, this ratio was close to the expected value (approximately 2). In sample 1 Mo, the molar ratio was higher than those of 2 Mo and 3 Mo. This indicates an increased amount of sulfur. This may be due to sulfur adsorption on the carbon support’s surface during synthesis, where the presence of adsorbed sulfur prevented its complete removal during the filtration and purification of the resulting suspension. In addition, this indicates fully adsorbed MoS_2_ on the surface of the carbon support.

Table 2 presents the percentage composition of the ReS_2_/Vulcan sample. Carbon was also the main element in samples 1 Re, 2 Re, and 3 Re. The carbon content in the samples was proportional to the amount of carbon added at the beginning of the synthesis.

Table 2 shows the molar ratio S to Re in all of the studied samples. This molar ratio was correct for all samples and was about 2. Due to the slower reaction kinetics in rhenium disulfide synthesis compared with molybdenum disulfide, the resulting particles can be more thoroughly distributed and integrated along the carbon support surface. The slight excess of sulfur in compounds 1 Re and 2 Re may indicate superior adsorption of pure sulfur, which is formed in the reaction on the surface of the carbon support. Therefore, it could not be sufficiently washed out in the filtration process.

#### 2.1.3. X-Ray Powder Diffraction

**Figure 2:**
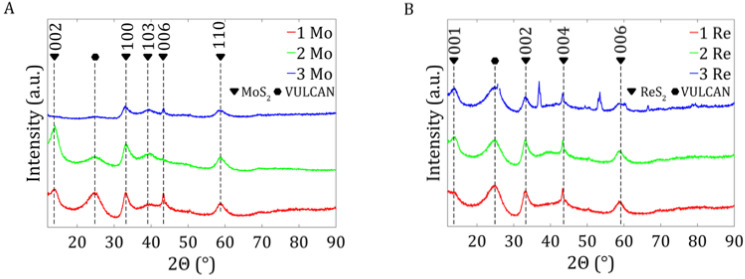
XRD patterns of samples 1 Mo, 2 Mo, 3 Mo for MoS_2_/Vulcan XC72R (**A**) and 1 Re, 2 Re, 3 Re for ReS_2_/Vulcan XC72R (**B**).

The diffractograms of the MoS_2_/Vulcan and ReS_2_/Vulcan samples are shown in Figure 2. All major diffraction peaks in Figure 2A corresponded to the standard hexagonal structure of 2H-MoS_2_ (JCPDS No. 37-1492), confirming the high purity of the samples obtained. The observed additional peak, marked with a hexagon in the diffractograms, corresponded to the carbon support Vulcan XC72R [20]. The intensity and width of the MoS_2_ peaks suggest differences in the degree of crystallinity and crystallite size between the samples studied. In particular, changes in peak intensity (002) may indicate a different degree of layered ordering of the material, and the presence of broad bands signals the involvement of an amorphous phase [21].

Figure 2B shows the standard peak pattern for ReS_2_ (JCPDS 82-1379), which demonstrates the high purity of the samples. No peak shifts were observed relative to the standard JCPDS chart, suggesting no significant stresses in the material’s crystalline structure. As with the MoS_2_ samples, an additional peak appeared for carbon [20].

For the 3Re sample, additional diffraction peaks were observed at 35°, 54°, and 67°, which can be attributed to the presence of the ReO_3_ phase. These peaks are consistent with the literature data for rhenium(IV) oxide [22] and were not present in the other samples analyzed, indicating their unique nature in this case. The presence of these reflections suggests that trace amounts of ReO_3_ may be present in the sample, most likely resulting from the partial oxidation of ReS_2_ during the baking process. Such a process may have been facilitated by a limited amount of carbonaceous material (Vulcan), which in larger amounts could act as a protective barrier or reductant, limiting oxygen access and thus reducing the risk of rhenium sulfide oxidation. As a result, the small presence of ReO_3_ in the 3Re sample seems reasonable from the point of view of the synthesis conditions and mixture composition.

The crystallite size was calculated based on the Scherrer equation and is presented in Table 3 and Table 4. The results for three different planes are presented to determine the crystallite sizes and shapes. The data showed that the crystallites in samples 1 Mo and 3 Mo had a cuboid shape, as one of their edges was significantly longer. In contrast, the crystallites in sample 2 Mo had a more cubic and uniform shape, as the edge lengths on each plane were similar. The ReS_2_ samples exhibited comparable crystallite sizes, with a notably shorter edge on the (001) crystallographic plane.

#### 2.1.4. Scanning Transmission Electron Microscopy

Comprehensive morphological and structural studies were carried out by STEM (scanning transmission electron microscope) for samples 1 Mo, 2 Mo, and 3 Mo containing MoS_2_ and samples 1 Re, 2 Re, and 3 Re with ReS_2_. The microstructural analysis was used to assess features influencing surface area including the particle size, crystallinity, and distribution of catalyst particles on the carbon support. The images obtained in secondary electron (SE) mode allowed for observations of the surface and morphology of the samples, while bright-field scanning transmission electron microscopy (BF-STEM) mode allowed for the assessment of their homogeneity. Images obtained at high resolution confirmed the crystalline form of the studied samples, indicating the degree of atomic structure ordering. Analysis of the distribution of MoS_2_ and ReS_2_ on Vulcan showed whether the active materials were uniformly deposited, which is crucial for their catalytic properties.

**Figure 3:**
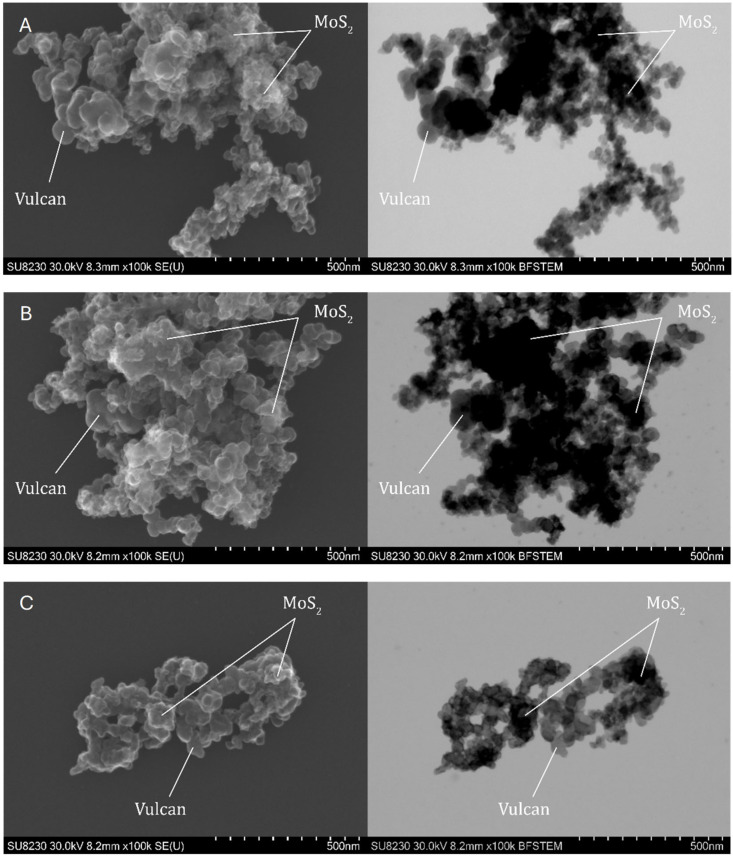
SEM and STEM images of samples 1 Mo (**A**), 2 Mo (**B**), and 3 Mo (**C**) for MoS_2_/Vulcan.

Analyzing the obtained images shown in Figure 3, it can be seen that samples 1 Mo and 2 Mo were evenly covered with MoS_2_ on most of the surface of the carbon support, indicating an effective deposition process of the active material. In addition, a large number of active centers were visible on the surface of the sample, which can favorably affect its electrochemical properties. Sample 3 Mo showed particles with single large agglomerates on them, which confirmed the sizes of the crystals obtained by XRD. The nonuniform distribution of active material on this sample may result in local variations in electrochemical activity, which is not desirable for applications requiring stable and well-dispersed active centers.

**Figure 4:**
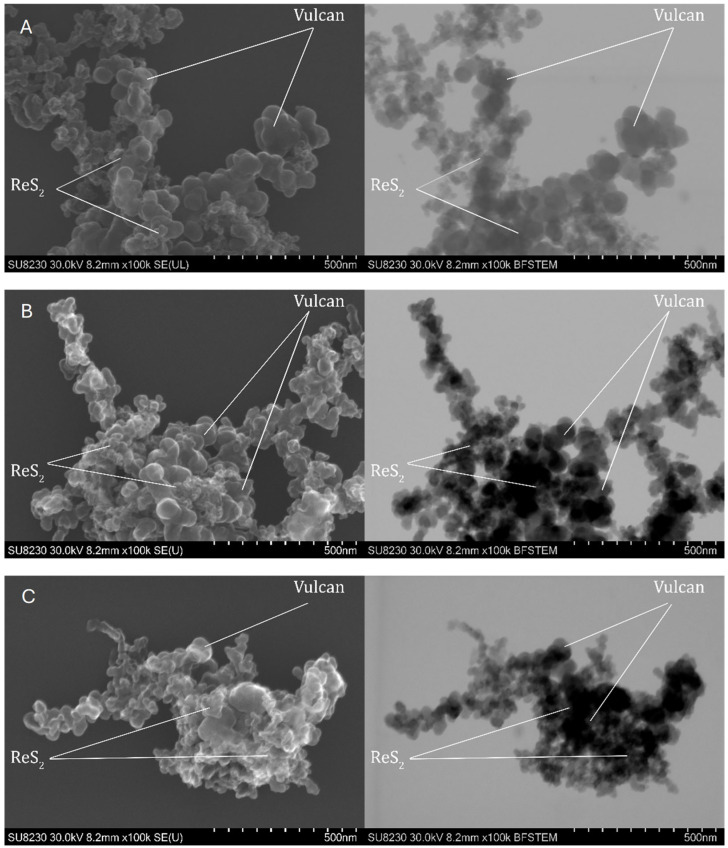
SEM and STEM images of samples 1 Re (**A**), 2 Re (**B**), and 3 Re (**C**) for ReS_2_/Vulcan.

Analyzing the images in Figure 4, it is clear that the 1 Re sample was widely heterogeneous. It showed clusters of agglomerates—large crystals—which was confirmed by the results of the XRD analysis. In contrast, samples 2 Re and 3 Re were more homogeneous. Their surface was covered with numerous small crystals, distributed as evenly as possible throughout the particle, suggesting a more homogeneous structure. There were larger clusters of ReS_2_ in sample 2 Re, indicating some degree of material aggregation. These results are consistent with previous XRD analyses, confirming differences in the structure and distribution of particles in the samples studied.

**Figure 5:**
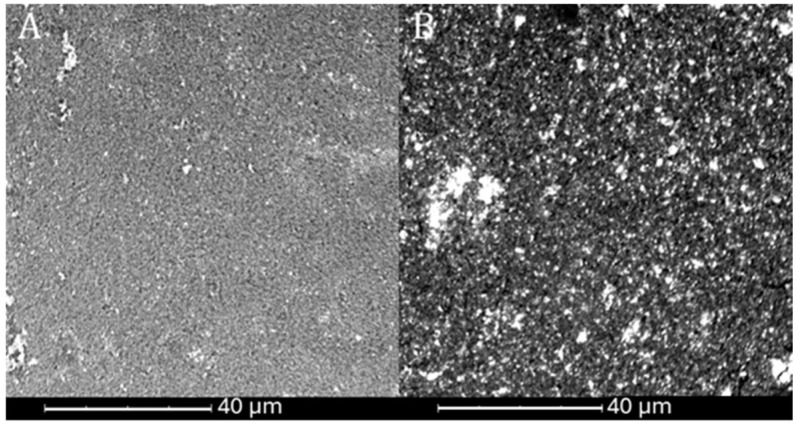
SEM images of MoS_2_/Vulcan (**A**) and ReS_2_/Vulcan (**B**) on carbon cloth.

After analyzing the SEM images of the carbon lattices with the catalyst applied, a uniform distribution of the catalyst layers could be observed. In the case of the MoS_2_/Vulcan material (Figure 5A), a homogeneous surface structure was observed, with the presence of fine grains indicating small agglomerates. The bright spots, corresponding to clusters of MoS_2_/Vulcan, were evenly distributed. The absence of a visible carbon lattice structure suggests coverage by a substantial and continuous layer of catalyst. In addition, the scale-like morphology characteristic of MoS_2_/Vulcan was noticeable.

In the case of ReS_2_/Vulcan (Figure 5B), the surface structure was clearly granular and irregular, with larger grains than in MoS_2_/Vulcan, which may indicate the presence of larger agglomerates, potentially resulting from the lower Vulcan content of the sample. The bright areas, which were clusters of ReS_2_/Vulcan, showed higher secondary electron reflectance, which may suggest a higher electron density. This catalyst was present as dense, heterogeneous nanoparticles forming clusters. As in the case of MoS_2_/Vulcan, the lack of a visible lattice indicates full surface coverage of the active material.

### 2.2. Electrochemical Performance

#### 2.2.1. Linear Sweep Voltammetry

The HER activity of the obtained MoS_2_/Vulcan and ReS_2_/Vulcan materials was studied using linear sweep voltammetry and the results were compared with those of samples without carbon nanomaterials. Figure 5 shows the results for MoS_2_/Vulcan and ReS_2_/Vulcan in different quantities of carbon.

**Figure 6:**
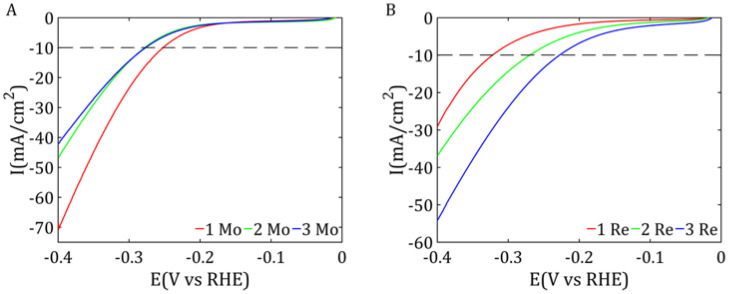
LSV curves normalized to the content of MoS_2_/Vulcan (**A**) and ReS_2_/Vulcan (**B**).

After analyzing the graphs presented in Figure 6 and the values in Table 5, it was observed that the 1 Mo sample exhibited the highest catalytic activity. A comparison of overpotential at a current density of −10 mA/cm^2^ showed that the lowest value was obtained for the 1 Mo sample. Samples 2 Mo and 3 Mo showed comparable but significantly lower activity than sample 1 Mo. This may have been due to less dispersion of MoS_2_ on the surface of the support or limited access to the active catalytic sites. The lower carbon content may result in the greater aggregation of MoS_2_ particles, reducing the active surface area and deterioration in electrical conductivity. Compared with the literature data, the results for sample 1 Mo were comparable to other samples on carbon [14,23] and were much better than pure MoS_2_ [24]. For samples based on ReS_2_, the best electrochemical activity was characterized by sample 3 Re. For a current density of −10 mA/cm^2^, the overpotential was −0.23 V. This value was lower than for sample 1 Mo, but sample 1 Mo achieved lower current density values at more negative potential values; this value was lower than for sample 1 Mo, but sample 1 Mo achieved higher current densities at lower potentials, indicating its better catalytic activity in the analyzed range. The worst results were obtained for sample 1 Re (−0.32 V). The results are consistent with the size of the crystals shown in Table 5. Sample 1 Re contained the largest crystals, which correlates with its relatively low electrochemical activity. On the other hand, sample 3 Re had the smallest crystals, giving it the best electrochemical activity. The results for the best sample are comparable to the literature results for ReS_2_ [25], where for −10 mA/cm^2^, the overpotential was −0.23 V.

#### 2.2.2. Cyclic Voltammetry

**Figure 7:**
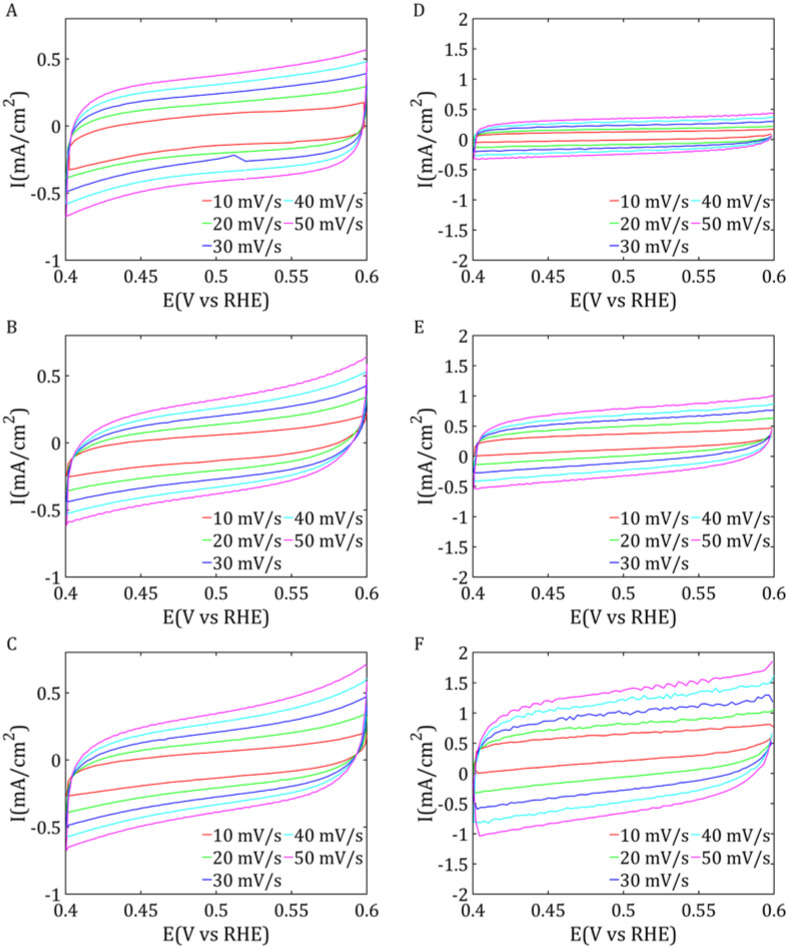
CV curves for MoS_2_/Vulcan (**A**) 1 Mo, (**B**) 2 Mo, and (**C**) 3 Mo and ReS_2_/Vulcan (**D**) 1 Re, (**E**) 2 Re, and (**F**) 3 Re.

To determine the capacitance of the double layer (C_dl_), CVA (cyclic voltammetry) measurements were conducted to increase the scanning speeds (10, 20…50 mV/s). The results of the measurements are shown in Figure 7. To determine the capacitance of the double layer, a potential range was chosen in which no redox reactions occur. At the most stable point in this range, the difference between the anodic and cathodic currents was calculated and then divided by two to obtain the value of the capacitor current. This measurement was repeated for each scan rate used. On this basis, a graph of the dependence of the capacitor current on the scanning rate was drawn, and the slope of the obtained straight line was taken as the value of the capacitance of the double layer. The calculation results are shown in Table 6.

For the MoS_2_-based materials, sample 1 Mo showed the highest C_dl_ value, reaching 8.2 mF/cm^2^, suggesting the largest active surface area among the samples analyzed. The other samples, 2 Mo, 3 Mo, and 1 Re, showed values of 7.4 mF/cm^2^, 7.7 mF/cm^2^, and 6 mF/cm^2^, respectively, indicating their similar electrochemical properties. Greater differences in C_dl_ values were observed for the ReS_2_ samples. Sample 3 Re showed the highest double-layer capacitance, reaching 18.9 mF/cm^2^, suggesting a much larger electrochemical surface area than the other samples. The largest active surface area was due to the greater number of active sites that had formed on the surface of the catalyst.

#### 2.2.3. Electrochemical Impedance Spectroscopy

**Figure 8:**
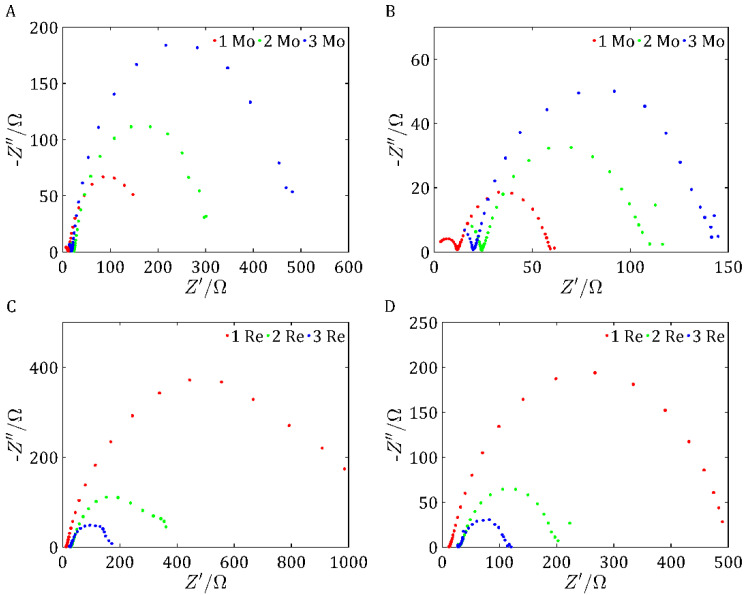
Nyquist spectra of samples based on MoS_2_ for an applied voltage of −0.4 V (**A**) and an applied voltage of −0.45 V (**B**), and samples based on ReS_2_ for an applied voltage of −0.4 V (**C**) and an applied voltage of −0.45 V (**D**).

Figure 8A shows the impedance values for the MoS_2_/Vulcan samples at an applied voltage of −0.4 V, while Figure 8B illustrates the exact measurements taken at −0.45 V. Analyzing the results, it can be seen that in both cases, sample 1 Mo had the lowest resistance values. A lower impedance means better charge transport and lower energy losses in the electrochemical system. This suggests a higher electrical conductivity and better electrocatalytic activity of sample 1 Mo compared with the other materials tested. In addition, the differences in impedance values between the samples remained consistent for both tested voltages, indicating the stability of the electrochemical properties of sample 1 Mo under different operating conditions.

Figure 8C presents the impedance values for the ReS_2_ samples at an applied voltage of −0.4 V, whereas Figure 8D depicts the precise measurements recorded at −0.45 V. The highest resistances appeared in both cases for sample 1 Re, which may indicate its less favorable electrochemical properties. The lowest resistances appeared for sample 3 Re, which had better conductivity and the highest electrochemical activity at higher potentials. These results corresponded to the LSV test results and confirmed the highest electrochemical activity of the tested sample.

**Figure 9:**
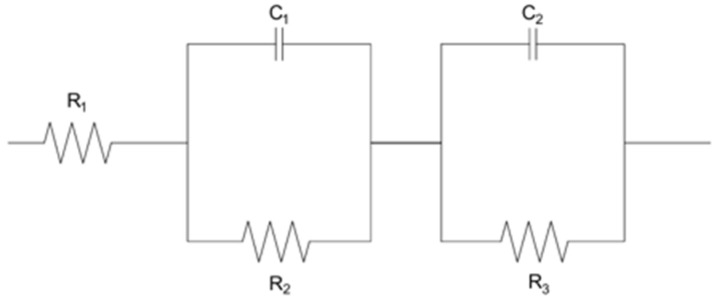
Electrical model of the electrochemical system for the EIS results.

The electrical model of the electrochemical system (Figure 9) under study consisted of a series resistance R1, which represents the resistance of the electrolyte, and two RC-type circuits connected in series. The first RC circuit, made up of the parallel connection of capacitor C1 and resistor R2, models the processes occurring on the electrode surface. In this circuit, capacitance C1 is responsible for the double layer, while resistance R2 represents the resistance associated with the charge transfer. These elements, together with the resistance R1, form the classical Randles model, corresponding to the first half-circle seen in the Nyquist diagram. Then, there is a second RC circuit, consisting of the parallel connection of resistor R3 and capacitor C2. This circuit reproduces an additional electrochemical process such as diffusion phenomena or the presence of a passive layer. The second RC circuit is responsible for the appearance of a second half-circle on the Nyquist diagram, characteristic of lower frequencies.

**Figure 10:**
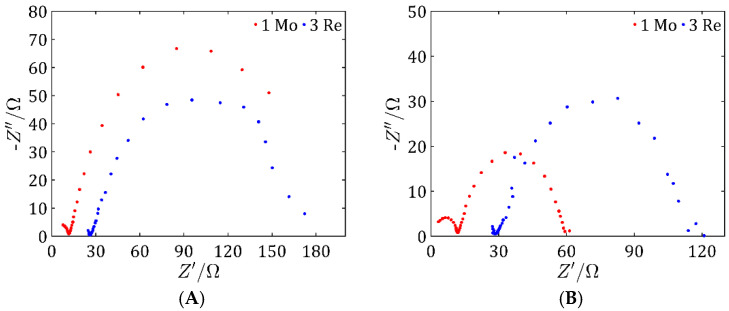
Nyquist spectra of samples 1 Mo and 3 Re for an applied voltage of −0.4 V (**A**) and an applied voltage of −0.45 V (**B**).

Figure 10 shows a comparison of the best sample for the MoS_2_-based catalysts and the best sample for the ReS_2_-based catalysts. Figure 10A shows the results for −0.4 V. For this voltage value, the lowest resistance was shown by sample 3 Re. On the other hand, for a potential of −0.45 V (Figure 10B), the lowest potential was shown by sample 1 Mo. Changes in resistance at different potentials may be due to dynamic electrochemical and material effects that affect the conductivity and efficiency of the electrocatalytic process.

#### 2.2.4. PEM Water Electrolysis

**Figure 11:**
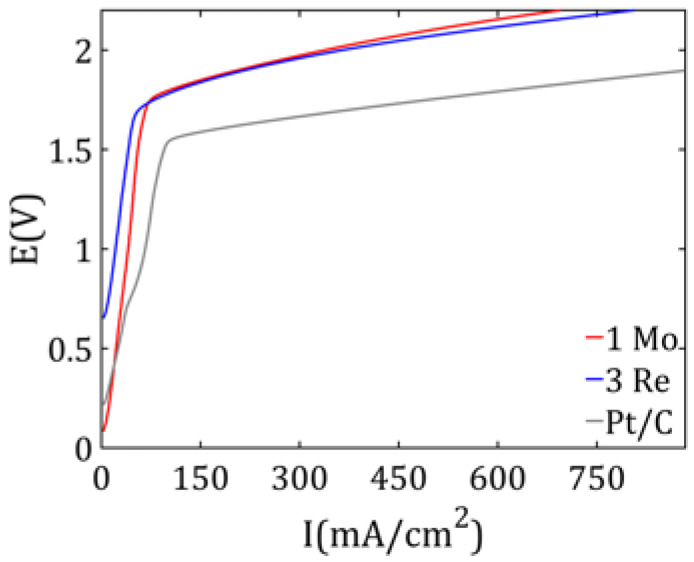
Polarization curves of PEM water electrolysis using 1 Mo, 3 Re, and Pt/C (reference) catalysts.

Analyzing the presented graph of potential (E) vs. current (I) dependence for 1 Mo, 3 Re, and reference Pt/C catalysts (Figure 11), it can be seen that catalysts based on MoS_2_ and ReS_2_ showed similar electrochemical activity. The results obtained were in agreement with the literature [10,26,27]. The two curves (red for 1 Mo and blue for 3 Re) almost overlapped, suggesting that these materials have comparable catalytic properties. For both materials, the amount of hydrogen produced was also measured for a constant intensity of 2 A. However, compared with platinum (gray curve), both MoS_2_ and ReS_2_ required a higher potential to achieve the same current. This means that their catalytic activity for PEM electrolysis is lower than that for Pt, which was expected, since platinum is one of the best commercial catalysts for this process. Nevertheless, the slightly higher energy requirements observed with MoS_2_ or ReS_2_ are not a critical drawback. The key advantage is the much lower cost of the material—platinum is rare and expensive, while molybdenum and rhenium are more common and are cheaper. As a result, despite the marginal increase in energy consumption, the overall reduction in the electrolyzer production costs makes these materials a promising and cost-effective alternative for large-scale hydrogen generation.

Table 7 presents the volumes of hydrogen obtained over a 50-s period of electrolyzer operation at a constant current of 2 A. The highest hydrogen yield was observed for the electrode with sample 1 Mo as the catalyst, followed by the electrode containing sample 3 Re. The lowest hydrogen production was recorded for the electrode coated with Pt/C. The maximum mass of hydrogen was calculated based on Faraday’s law, using Equation (1).

(1) $$m=\frac{MIt}{nF}$$

where *M* is the molar mass of the substance being measured, *I* is the intensity of the current being set, *t*- is the duration of the measurement, *n*- is the number of electrons involved in the reaction that produces 1 mole of substance of molar mass *M*, and *F* is Faraday’s constant of 96485.33 C/mol.

After converting the calculated mass to volume at a temperature of 60 °C and a pressure of 1 atm, a value of 14.16 mL was obtained. The measured gas volume for molybdenum sulfide slightly exceeded the theoretical value. This may be due to measurement error or residual hydrogen remaining in the diffusion layer. Nevertheless, the results indicate that nearly 100% hydrogen evolution efficiency was achieved for MoS_2_ and ReS_2_. In contrast, the Pt/C catalyst appeared to exhibit a limiting factor affecting the reaction performance. A possible explanation for the reduced performance of the Pt/C electrode is the accumulation of hydrogen gas bubbles on the electrode surface. Excessive hydrogen evolution may have led to the blockage of flow channels, thereby limiting water access to the catalytic sites. As a result, the effective electrochemically active surface area could have been reduced, negatively impacting the overall hydrogen production efficiency [26,27].

## 3. Materials and Methods

### 3.1. Materials

This study utilized catalytic materials based on molybdenum disulfide and rhenium disulfide deposited on a nanocarbon support—Vulcan XC72R (purchased from Sigma-Aldrich, Tokyo, Japan). Three samples of MoS_2_ and three samples of ReS_2_ were synthesized via the wet chemical synthesis method, each differing in the amount of carbon support added.

This study aimed to determine the appropriate amount of carbon support and analyze its influence on key physicochemical and electrochemical properties of the tested catalysts. In particular, attention was given to the change in material structure, specific surface area, electrical conductivity, and catalytic activity in electrochemical processes.

### 3.2. MoS_2_ Synthesis Procedure

MoS_2_ was obtained by wet synthesis as described in [13,14]. The process was carried out using a solution of ammonium sulfide NH_4_S (AS) (purchased from Chempur, Piekary Śląskie, Poland) and ammonium heptamolybdate (NH_4_)_6_Mo_7_O_24_·4H_2_O (HMA) (purchased from Supelco, Darmstadt, Germany) with citric acid C_6_H_8_O_7_ (CA) (purchased from Sigma Aldrich, Tokio, Tokyo, Japan) acting as the reducing agent and pH regulator of the reaction environment. Subsequently, three different amounts of carbon soot were selected for three consecutive samples based on previous research [14], with the specific quantities listed in Table 8.

The Vulcan XC72R support was introduced into the mixture of HMA and CA as a support for MoS_2_ particle growth in three different amounts labelled 1 Mo, 2 Mo, and 3 Mo. The synthesis reactions were carried out in an S-type jet reactor, which facilitates homogeneous mixing and controlled reaction conditions. The resulting product was filtered using 45 µm pore filters. The resulting precipitate was dried and annealed in a furnace at 550 °C under argon flow for one hour.

### 3.3. ReS_2_ Synthesis Procedure

ReS_2_ was obtained by wet chemical synthesis, as described in [15]. For this purpose, a solution of sodium sulfide Na_2_S (≥98.0%, Chempur, Piekary Śląskie, Poland) and a mixture of ammonium perrhenate NH_4_ReO_4_ (NRA) (≥99.9%, Innovator, Gliwice, Poland) with formic acid HCOOH (85%, p.a., POCH, Gliwice, Poland) was used. Vulcan XC72R was added in three different amounts labelled 1 Re, 2 Re, and 3 Re to the NRA and FA solution. The amounts of carbon support added are shown in Table 9. The synthesis reactions were carried out in an S-type jet reactor. As the reaction does not occur immediately, the resulting mixture was additionally stirred on a magnetic mixer for 15 min to ensure complete homogeneity. Like MoS_2_, the resulting product was filtered using 45 µm pore filters, dried, and annealed in a furnace at 550 °C under argon flow for one hour.

### 3.4. Physicochemical Characteristics

The study presented two types of catalysts with different carbon contents in the samples. To determine the physicochemical properties affecting the electrochemical properties of the material, a comprehensive material analysis was carried out using various analytical techniques such as thermogravimetric analysis (TGA), X-ray diffraction (XRD), X-ray fluorescence (XRF), and scanning electron microscopy (STEM).

Thermogravimetric analysis was conducted to determine changes in the mass of the material as a function of temperature, which allowed for the identification of thermal processes such as decomposition, oxidation, evaporation, or thermal stability. For this purpose, a TGA/DSC 3+ analyzer from Metler Toledo was used. The tests were conducted from 30 °C to 1200 °C with a temperature change of 10 °C/min with 60 mL/min airflow.

The X-ray fluorescence analysis was conducted to identify and quantify the elements present in the studied samples. The measurements were conducted using an Epsilon 3XLE by PANalytical instrument (Zevenhuizen, The Netherlands). Samples were analyzed in special containers with a diameter of 28 mm, in which the bottom was a film made of a permeable X-ray polymer (Mylar) with a thickness of 3.6 µm. The Mo:S and Re:S ratios were determined, allowing for the verification of the material’s stoichiometry. This method uses the characteristic emission of radiation by atoms excited by X-rays.

X-ray powder diffraction analysis determined the phase composition of the MoS_2_ and ReS_2_ samples under study. For this purpose, we used a Anton Paar XRDynamic 500 diffractometer with a copper lamp (40 kV, 40 mA) and Primux 3000 detector. The results were analyzed using the Fityk program [28]. The crystallite sizes were calculated using the Sherrer equation in Equation (2), where λ is the X-ray wavelength, L is the integral width of the Bragg reflex, and θ is the Bragg angle.

(2) $$\beta (2\theta )=\frac{\lambda }{L\;cos\theta }$$

Observations of the topography and structure of the powders were carried out using a Cs-corrected scanning transmission electron microscope (STEM), model Hitachi SU8230 (Naka, Japan). Samples were prepared by suspending the powder in isopropanol, after which four drops of 10 µL each were deposited onto a copper TEM grid. The observations were performed in two modes: topographic using secondary electron (SE) and diffraction contrast for high-resolution bright field images (BF).

### 3.5. Electrochemical Performance

A series of electrochemical tests were conducted to investigate the potential of the MoS_2_- and ReS_2_-based samples as catalysts for the HER. For this purpose, linear sweep voltammetry (LSV), cyclic voltammetry (CVA), and electrochemical impedance spectroscopy (EIS) were used. Analyses were performed using a three-electrode system connected to a SP-200 potentiostat (BioLogic, Seyssinet-Pariset, France). The electrochemical system used a glassy carbon electrode (GCE) with an active surface of 0.071 cm^2^, an auxiliary electrode made of Pt, and an Ag/AgCl reference electrode saturated with 3 M NaCl solution as the working electrode. The experiments were conducted in 0.5 M sulfuric acid (VI), providing adequate ionic conductivity in the reaction system.

Catalytic inks were prepared by weighing 2 mg of each catalyst, then adding 450 µL of isopropanol and 50 µL of Nafion solution (alcohol-based, 1000 ew at 5 wt.%, Ion Power, Germany, Munich). Isopropanol acted as the solvent, while Nafion acted as the binder to ensure the adhesion and homogeneity of the ink. The prepared mixtures were ultrasonicated for 30 min to obtain homogeneous suspensions. Next, 10 µL of the resulting catalytic ink was deposited onto the surface of a glassy carbon electrode and allowed to dry at room temperature. To remove dissolved oxygen and create an inert reaction atmosphere, the entire system was flushed with argon for 30 min before the measurements.

The LSV measurements were conducted in a voltage range from −0.4 to 0 V vs. RHE (reversible hydrogen electrode) at a scan rate of 50 mV/s, allowing for the analysis of electrochemical reactions occurring on the surface of the catalytic material within this potential range. Tafel slopes were calculated from the LSV measurements based on Equation (3), where *η* is the overpotential, *A* is the “Tafel slope”, *i* is the current density, and *i_0_* is the exchange current density.

(3) $$\eta =A\cdot log\left(\frac{i}{i_{0}}\right)$$

The cyclic voltammetry results can be used to calculate the double-layer capacitance (*C_dl_*), which is directly related to the electrochemically accessible surface area (ECSA) of the material. The value of *C_dl_* can be determined based on the linear relationship between the capacitive current and the scan rate. A higher *ECSA* value indicates a greater number of active sites for electrochemical processes, which is crucial for the catalyst efficiency. The value of ECSA is linearly proportional to *C_dl_* based on Equation (4).

(4) $$ECSA=\frac{C_{dl}}{C_{s}}$$

*C_s_* is a specific capacitance; it is a characteristic value depending on the type of electrode used for the experiment. The literature shows that this value is often between 20 and 60 μF/cm^2^.

EIS measurements were carried out at frequencies of 200 kHz to 50 mHZ at −0.4 V and −0.45 V to understand the processes occurring at the electrode–electrolyte interface and to evaluate the electrochemical properties of the materials studied. The technique provides detailed information on the resistances, capacitances, and electrochemical reaction mechanisms.

### 3.6. PEM Water Electrolysis

In order to evaluate the performance of the tested catalysts in PEM electrolysis, an iridium oxide-based anode and a cathode were prepared using the analyzed materials.

The anodic ink was deposited on a 6.76 cm^2^ titanium fiber mesh. The catalytic ink contained IrO_2_ at 2 mg/cm^2^, and 18 mg of catalyst was used to prepare it, taking into account a 30% loss when applying the ink to the mesh. Then, 200 µL of water and 40 µL of Nafion solution were added.

The cathodes were prepared so that the catalyst content was 0.4 mg/cm^2^—analogous to the amount of Pt/C used in commercial applications (purchased from Fuel Cell Store, Bryan, TX, USA). The ink was applied to a carbon cloth with the same surface area as the anode. Next, 20 mg MoS_2_/Vulcan (assuming 17% MoS_2_ content in the sample and 30% material loss) and 7.38 mg ReS_2_/Vulcan (assuming 44% ReS_2_ content in the sample and 30% material loss) were used. Subsequently, 200 µL of water was added to both catalysts, 200 µL for MoS_2_, and 75 µL for ReS_2_ of a 5% Nafion solution. To obtain the reference potential curves, a platinum-based cathode (Pt/C) was prepared. A total of 9 mg of powder containing 40% Pt was used, to which 200 µL of water and 91 µL of 5% Nafion solution were added.

Before application to the electrodes, the catalytic carcasses were sonicated for more than one hour in order to obtain as homogeneous a suspension as possible. The prepared inks were applied to the grid using a brushing method, commonly described in the literature as an effective technique for preparing electrodes for cells [29,30]. The electrodes were then annealed at 130 °C under pressure. The membrane used in the study was Nafion 117 (Ion Power, Munich, Germany) with a thickness of about 183 µm, which is a synthetic copolymer of tetrafluoroethylene and perfluorinated oligovinyl ether terminated with strongly acidic sulfonic groups. The membrane comes in the form of a thin, flexible film due to its high proton conductivity and exceptional chemical and thermal resistance. The membrane was placed in a PEM electrolyzer, which used bipolar plates with serpentine flow channel geometry to ensure the uniform distribution of deionized water supplied at a flow rate of 500 mL/min as well as the efficient removal of gaseous reaction products.

To compare the hydrogen production efficiency, measurements were conducted for all three prepared cathodes. According to Faraday’s law [31], which states that the amount of an electrochemically deposited substance is proportional to the applied current and the duration of electrolysis, all experiments were carried out at a constant current of 2 A. The hydrogen that evolved during the electrolysis process was collected in a water-filled graduated cylinder submerged in a water bath. The volume of collected hydrogen was recorded after 50 s of electrolyzer operation.

## 4. Conclusions

In this study, we presented MoS_2_- and ReS_2_-based catalysts supported on a carbon nanomaterial, synthesized via a wet-chemical method using a continuous-flow impinging jet reactor. The use of this type of reactor enabled precise control over the synthesis conditions, resulting in high reproducibility and uniformity of the obtained materials.

Comprehensive physicochemical and electrochemical characterizations were carried out for the synthesized catalysts. The results indicate that the most promising samples were sample 1 Mo among the MoS_2_-based catalysts and sample 3 Re among the ReS_2_-based ones. These findings are supported by structural analyses including SEM imaging, which revealed a highly developed active surface area of the catalyst on the Vulcan carbon support, suggesting a large number of catalytically active sites.

Due to this extensive surface area, these samples also exhibited the best electrochemical properties, as evidenced by the low overpotentials in the LSV measurements, which were comparable for both samples. In addition, both catalysts demonstrated high double-layer capacitance, particularly pronounced for ReS_2_, and low impedance values, indicating efficient charge transfer characteristics.

Finally, the catalysts were evaluated as cathodic materials. Both exhibited an electrochemical performance comparable to platinum, with a slightly lower efficiency. However, given the significantly lower cost of these catalyst materials, the performance difference is minor from a practical standpoint. This study highlights the great potential of rhenium and molybdenum as cost-effective alternatives to platinum in electrochemical catalysis, particularly for hydrogen evolution reactions.

## Figures and Tables

**Table 1:** Percent composition of the MoS_2_/Vulcan sample and molar ratio S to Mo in the MoS_2_/Vulcan samples.

	1 Mo	2 Mo	3 Mo
Mo [%]	10.2	44.5	33.5
S [%]	6.4	13.9	12.0
C [%]	83.4	41.6	54.5
S/Mo	4.1	1.4	1.8

**Table 2:** Percent composition of the ReS_2_/Vulcan sample and molar ratio S to Re in the ReS_2_/Vulcan samples.

	1 Re	2 Re	3 Re
Re [%]	9.5	17.7	34.0
S [%]	3.9	6.1	9.7
C [%]	86.6	76.2	56.3
S/Mo	2.3	2.7	2.0

**Table 3:** Average crystallite size for MoS_2_/Vulcan.

Plane	(002)	(100)	(006)
1 Mo	5 nm	3 nm	11 nm
2 Mo	4 nm	4 nm	4 nm
3 Mo	4 nm	6 nm	17 nm

**Table 4:** Average crystallite size for ReS_2_/Vulcan.

Plane	(001)	(002)	(006)
1 Re	2 nm	5 nm	4 nm
2 Re	2 nm	4 nm	4 nm
3 Re	3 nm	4 nm	5 nm

**Table 5:** Overpotential at a current density of −10 mA/cm^2^.

	1 Mo	2 Mo	3 Mo	1 Re	2 Re	3 Re
Overpotential [V vs. RHE]	−0.25	−0.27	−0.27	−0.32	−0.27	−0.23

**Table 6:** The double layer capacitance for samples 1 Mo, 2 Mo, and 3 Mo and 1 Re, 2 Re, and 3 Re.

	1 Mo	2 Mo	3 Mo	1 Re	2 Re	3 Re
Capacitance [mF/cm^2^]	8.2	7.4	7.7	6.0	11.4	18.9

**Table 7:** Amount of measured hydrogen for each catalyst.

	1 Mo	3 Re	Pt/C
Hydrogen (mL)	15	14	10

**Table 8:** The amount of carbon support added to successive samples of MoS_2_.

Sample Name	Mass of Vulcan XC72R [mg]
1 Mo	305
2 Mo	71
3 Mo	43

**Table 9:** The amount of carbon support added to successive samples of ReS_2_.

Sample Name	Mass of Vulcan XC72R [mg]
1 Re	1400
2 Re	160
3 Re	100

## Data Availability

The data presented in this study are available on request from the corresponding author due to confidentiality agreements and proprietary restrictions related to ongoing industrial collaboration.
